# Supplementary material for: Disease transmission and introgression can explain the long-lasting contact zone of modern humans and Neanderthals
Source: Nat Commun. 2019 Nov 1;10:5003. doi: 10.1038/s41467-019-12862-7 (PMC6825168; doi:10.1038/s41467-019-12862-7)
Supplement: Supplementary file 1 — Supplementary Information [file 41467_2019_12862_MOESM1_ESM.pdf]

Supplementary Information

Disease transmission and introgression can explain the long-lasting contact zone of modern humans and Neanderthals

Greenbaum *et al.*

Table of Content

Supplementary Note 1 — Exploration of the effects of model parameters 2

Supplementary Note 2 — Modeling regular adaptation in addition to adaptive introgression 18

Supplementary Note 3 — Modeling inter-species competition 23

Supplementary Note 1 — Exploration of the effects of model parameters

In this section, we explore several of the parameters of the models. The goal of this section is not an exhaustive exploration of the parameter space, but rather to demonstrate that the dynamics are qualitatively similar for a significant portion of this space, in the sense that the three phases described in the main text (Figures 3–5 in the main text) can be observed. It is also aimed at providing some intuition about how the parameters control the dynamics, which we summarized as the time of onset of the second and third phases. We do not conduct this analysis for all the parameters in the models, but rather focus on a few which we believe such an analysis can provide insights.

For the two-time-scales model (Equations (1)–(15) in the main text), we explore varying initial (which is also intrinsic) between-species contact rates ($\beta_{12}(0)$ and $\beta_{21}(0)$), the ratio between disease transmission and introgression (c), and initial asymmetry in within-species contact rates ($\beta_{11}(0) \neq \beta_{22}(0)$). We do not conduct the analysis for the scaling parameters (a_{ij} and b_{ij}) which adjust the relative impacts of disease burden and tendency to return to initial conditions, since we vary the other parameters which determine disease burden. We also do not conduct the analysis for the recovery rate γ, which affects disease burden in a fairly straightforward way (longer recovery times amount to more infected individuals and higher F_i in Equations 9 in the main text).

For the single-time-scale model, we explored varying between-species contact rates (β_a), the ratio between disease transmission and introgression (c), and initial asymmetry in intrinsic population densities ($N_1(0) \neq N_2(0)$). Since we focus on the disease and introgression processes, we do not vary the demographic parameters λ, K and μ. For tractability, we vary one parameter at time, using the parameters presented in the main text as a baseline.

Two-time-scales model

In order to compare the dynamics over several parameter ranges, we summarized the dynamics using two values, the time of onset of phase II, and the time of onset of phase III (Figures 3 and 5 in the main text). For the time of onset of phase II, we first defined for each of the contact rate parameters the time point, $u_{\beta_{ij}}$, as the first time-step in which the absolute difference between β_{ij} at consecutive time steps was lower than 10^{-3}; in other words, $u_{\beta_{ij}}$ was defined as the lowest t for which $|\beta_{ij}(t) - \beta_{ij}(t-1)| < 10^{-3}$. This is a discrete equivalent for finding the first time point in which the first derivative of the contact rate is very close to 0, indicating arrival at the stable phase. The threshold (10^{-3}) was selected so as not to be too

small and miss the stabilization of the dynamics at phase II, but not too high as to define stability when the slope was still observably significantly different then 0. The overall onset of phase II, u, was taken to be the maximum over the four $u_{\beta_{ij}}$ values (for $i, j = 1, 2$), meaning that we consider the onset of phase II as the first time step in which all contact rates are considered to have arrived at the steady state.
The time of onset of phase III, w, was defined as the first time step for which disease burden in either species was removed; in other words, the earliest t for which either $D_1(t) = 0$ or $D_2(t) = 0$.

Between-species contact rates

We first varied the between-species contact rates, which directly affects both the transmission of disease in the system and the rate of adaptive introgression. We used the parameter values in Figures 3 and 4 in the main text as a baseline, and varied β_{ij} ($i \neq j$) from 0.005 to 0.03 in increments of 0.001 (maintaining $\beta_{ij} = \beta_{ji}$). For between-species contact rates below 0.005, the numerical solutions of the differential equations in Equations (3)–(8) in the main text were affected by computational precision errors due to very low values, and we therefore restricted our range to avoid these low ranges of transmission. We conducted this analysis for the symmetric ($P_1(0) = P_2(0)$) and asymmetric cases ($P_1(0) \neq P_2(0)$), as presented in the main text.
Supplementary Figure 1 shows the time of onset for the two phases for the different within-species contact rates. We also show the full dynamics over time for two examples, one with between-species contact rates higher than those shown in the main text ($\beta_{ij} = 0.2$, in blue) and one with lower rates ($\beta_{ij} = 0.05$, in green). This we do to demonstrate that the dynamics are qualitatively similar to the scenarios described in Figures 3 and 4 in the main text, in the sense that three distinct phases can be observed (Supplementary Figure 2).
The onset of phase II increases with increased between-species contact, for both the symmetric and asymmetric scenarios examined (Supplementary Figure 1A and 1B), while the onset of phase III decreases for both scenarios (Supplementary Figure 1C and 1D). Overall, since the changes in w are much larger than those in u, the length of Phase II ($w - u$) is more affected by w, and therefore, for the parameter range we examined, the length of phase II decreases with increased between-species contact. We also show two examples of the dynamics (Supplementary Figure 2), which show qualitatively similar dynamics to those in Figures 3 and 4 in the main text, but the dynamics occur over different times scales, with faster dynamics in the case of high between-species transmission (in blue) compared to that with low between-species contact rates (in green).
The between-species contact rate parameter β_{ij} plays two roles in the model: (1) Increase in β_{ij} is expected to increase F_i (Equations (1)–(8) in the main text), and

therefore increase disease burden, D_i (Equation 9 in the main text), which would lead to a decrease in all contact rates (Equations (10)–(13) in the main text);(2) Increase in β_{ij} increases the rate of introgression (Equations (14) and (15) in the main text). At the early stages of the dynamics, when the species are strongly impacted by epidemics, increased disease burden results in significant reductions in between-species contact,and therefore slower introgression, leading to later onset of phase II (Supplementary Figure 1A and 1B). However, once disease burden decreases over time, the high intrinsic $\beta_{ij}(0)$ leads to faster introgression, reducing the time to onset of phase III (Supplementary Figure 1C and 1D).

Ratio of introgression to disease transmission

The two-time-scales model assumes that both introgression and disease transmission are coupled with the between-species contact rates, with a fixed parameter describing the ratio between introgression and between-species contact, c. We varied c between 10 and 5000 in increments of 10, with the other parameters kept the same as those in Figures 3 and 4 in the main text.

Supplementary Figure 3 shows the time of onset for the two phases for the c values. We also show the full dynamics over time for two examples (Supplementary Figure 4), one with c value higher than the one described in the main text ($c = 1000$, in blue), and one with a lower c value ($c = 10$, in green). These are shown to demonstrate that the dynamics are qualitatively similar to the scenarios described in Figures 3 and 4 in the main text, in the sense that three distinct phases can be observed.

The onset of phase II is unchanged for up to about $c = 1000$, above which the onset time u decreases as the ratio c increases (Supplementary Figure 3). The onset of phase III decreases with high c values (Supplementary Figure 3), which is due to the fact that a high c value means faster adaptive introgression and faster overcoming of disease burden, in both the symmetric and asymmetric cases.

The ratio between disease transmission and introgression, c, plays a role in Equations (10)–(13) in the main text, where higher c values lead to faster introgression. Therefore, increasing c results both in earlier onset of phase II and earlier onset of phase III (Supplementary Figure 3).

Initial asymmetry in contact rates

In Figure 4 in the main text, we explore the consequences of initial asymmetry in pathogen package size, P. Here we consider initial asymmetry in the intrinsic within-species contact rate, $\beta_{11}(0)$ and $\beta_{22}(0)$. Such asymmetry could reflect, for example, differences in population densities (within-species contacts occur at lower rates in sparser populations), but also differences in contact tendencies of the two species. One

scenario where such differences may have occurred is if the incoming Moderns were characterized, over a long period, with lower population densities than the resident Neanderthals. However, this phase may have been a short transient phase for the Moderns, in which case it would not necessarily have been influential for the dynamics we consider.

To explore this source of asymmetry, we considered the symmetric case in Figure 3 in the main text as a baseline and varied the within-species contact rates for species 2, $\beta_{22}(0)$, from 0.005 to 0.05 at increments of 0.001, with the other parameters kept the same as those in Figure 4 in the main text (in particular, $\beta_{11} = 0.05$). Supplementary Figure 5 shows the time of onset for the two phases for the $\beta_{22}(0)$ values. We also show the full dynamics over time for two examples (Supplementary Figure 6), one with $\beta_{22}(0) = 0.03$ (in blue), and one with $\beta_{22}(0) = 0.015$ (in green).

With asymmetry in within-species contact rates, the time of onset of phase II is almost unchanged for the parameter range we investigated, but the onset of phase II is slightly sooner with more accentuated asymmetry (Supplementary Figure 5). Interestingly, and perhaps counterintuitively, with such asymmetry, species 2, which initially had lower within-species contact rates, overcomes disease burden sooner than species 1. This is because the lower within-species contact rates translate to lower F_2 values in Equations (1) and (2) in the main text. and hence lower initial disease burden (Supplementary Figure 6C and 6F), which allows it to maintain higher incoming between-species contact rates (Equations (10)–(13) in the main text, and Supplementary Figure 6A and 6D). These between-species contact rates are then translated to higher rates of adaptive introgression (Equations (14) and (15) in the main text, and Supplementary Figure 6B and 6E), and more rapid overcoming of disease burden.

The initial condition of β_{22} determines the intrinsic within-species contact rates in the species (Equations (10)–(13) in the main text). In the model, within-species contact rates play a role in determining the impact of diseases on the species (Equations (1)–(9) in the main text), but do not directly affect introgression. Therefore, decreasing the within-species contact rate of one species relative to the other, results in less impact of disease, allowing the species to maintain higher between-species contact rates and increases the rate of introgression. This leads to earlier onset of phase III with lower within-species contact rate in one of the species.

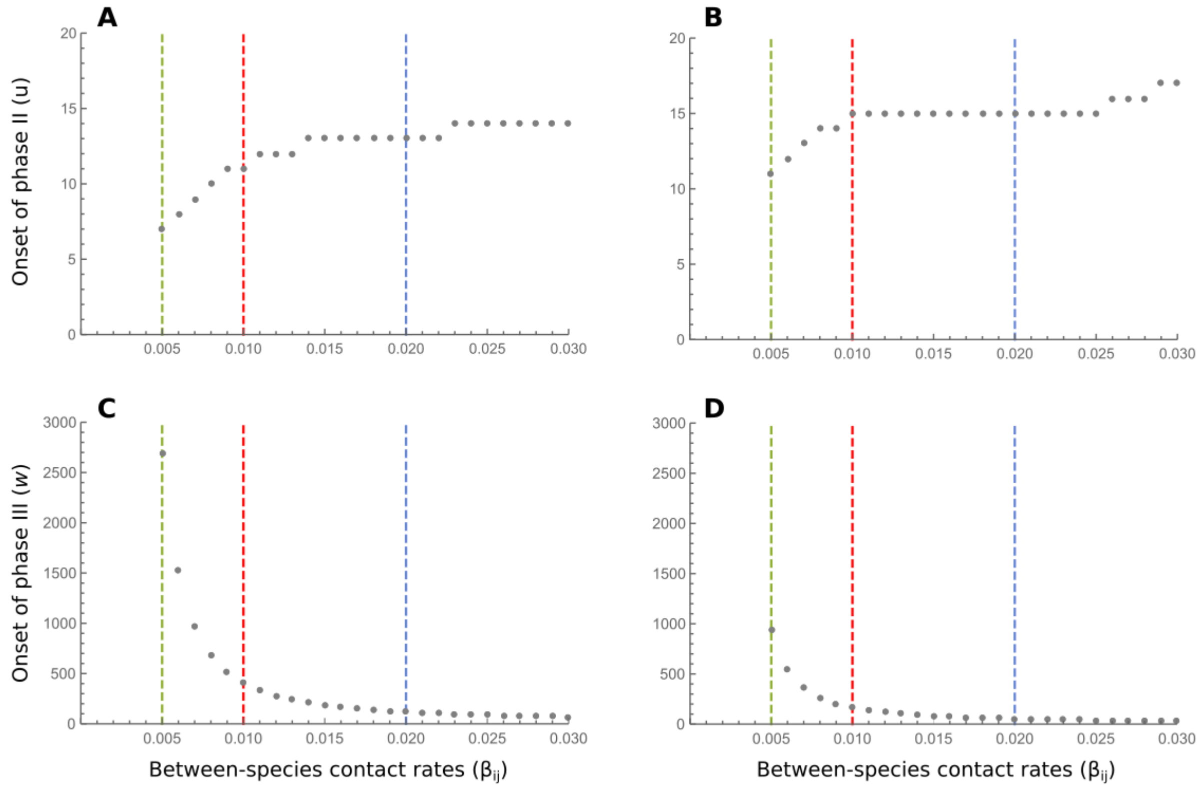

Supplementary Figure 1: Time of onset of phases II (u) and III (w) for different between-species contact rates ($\beta_{ij}(0)$) in the two-time-scales model. Dashed lines show the parameter values for which we show the dynamics explicitly, in the main text in Figures 3 and 4 (red), or in Supplementary Figure 2 (green and blue). (A) Onset of phase II for the symmetric case. (B) Onset of phase II for the asymmetric case ($P_1(0) = 60$, $P_2(0) = 100$). (C) Onset of phase III for the symmetric case. (D) Onset of phase III for the asymmetric case ($P_1(0) = 60$, $P_2(0) = 100$). Other than the the between-species contact rates, all other parameters are the same as those in Figure 3 (for (A) and (C)) and Figure 4 (for (B) and (D)) in the main text.

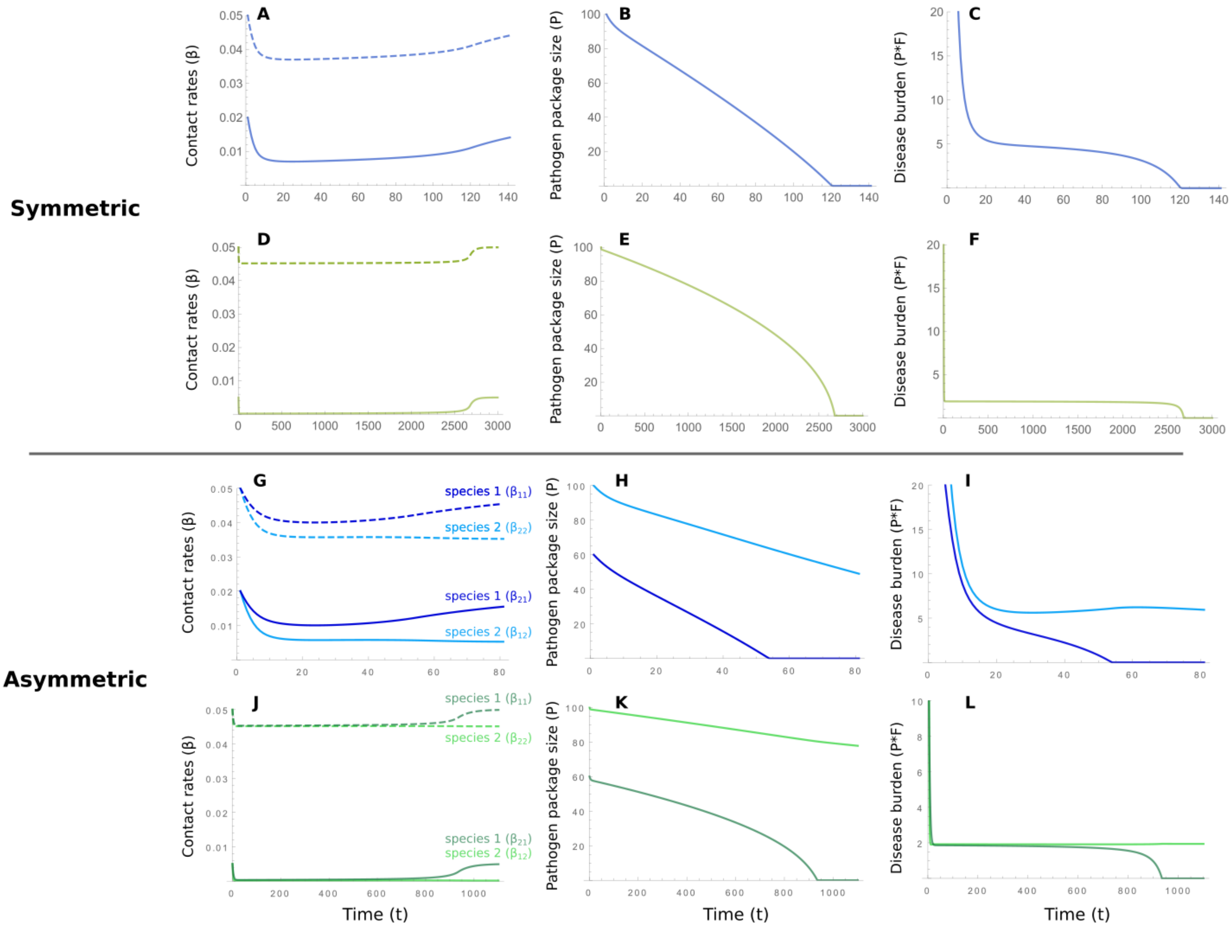

Supplementary Figure 2: Two examples of dynamics of the two-time-scales model for the values of between-species contact rates (β_{ij}) marked in Supplementary Figure 1. (A)–(F) show the results for the symmetric case, comparable with Figure 3 in the main text, and (G)–(L) show the results for the asymmetric case, comparable with Figure 4 in the main text. For the panels with blue curves (corresponding to the blue line in Supplementary Figure 1), between-species contact rates are $\beta_{ij}(0) = 0.02$, while the rest of the parameters are the same as those in Figures 3 and 4 in the main text. For the panels with green curves (corresponding to the green line in Supplementary Figure 1), between-species contact rates are $\beta_{ij}(0) = 0.005$, while the rest of the parameters are the same as those in Figures 3 and 4 in the main text. Notice that the times scales for the different panels are different.

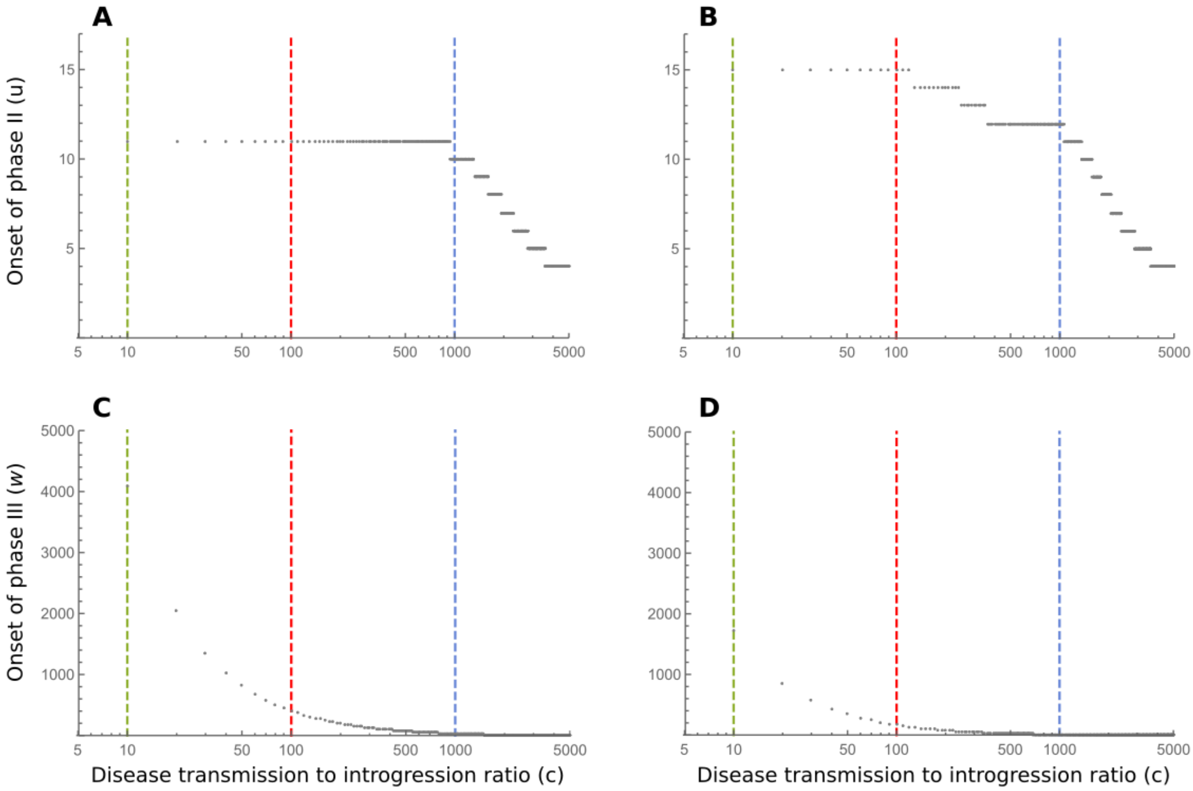

Supplementary Figure 3: Time of onset of phases II (u) and III (w) for different introgression to disease transmission ratios (c) in the two-time-scales model. Dashed lines show the parameter values for which we show the dynamics explicitly, in the main text in Figures 3 and 4 (red), or in Supplementary Figure 4 (green and blue). (A) Onset of phase II for the symmetric case. (B) Onset of phase II for the asymmetric case ($P_1(0) = 60$, $P_2(0) = 100$). (C) Onset of phase III for the symmetric case. (D) Onset of phase III for the asymmetric case ($P_1(0) = 60$, $P_2(0) = 100$). Other than the the between-species contact rates, all other parameters are the same as those in Figure 3 (for (A) and (C)) and Figure 4 (for (B) and (D))in the main text. Notice that the c parameter on the X-axis is plotted on a logarithmic scale.

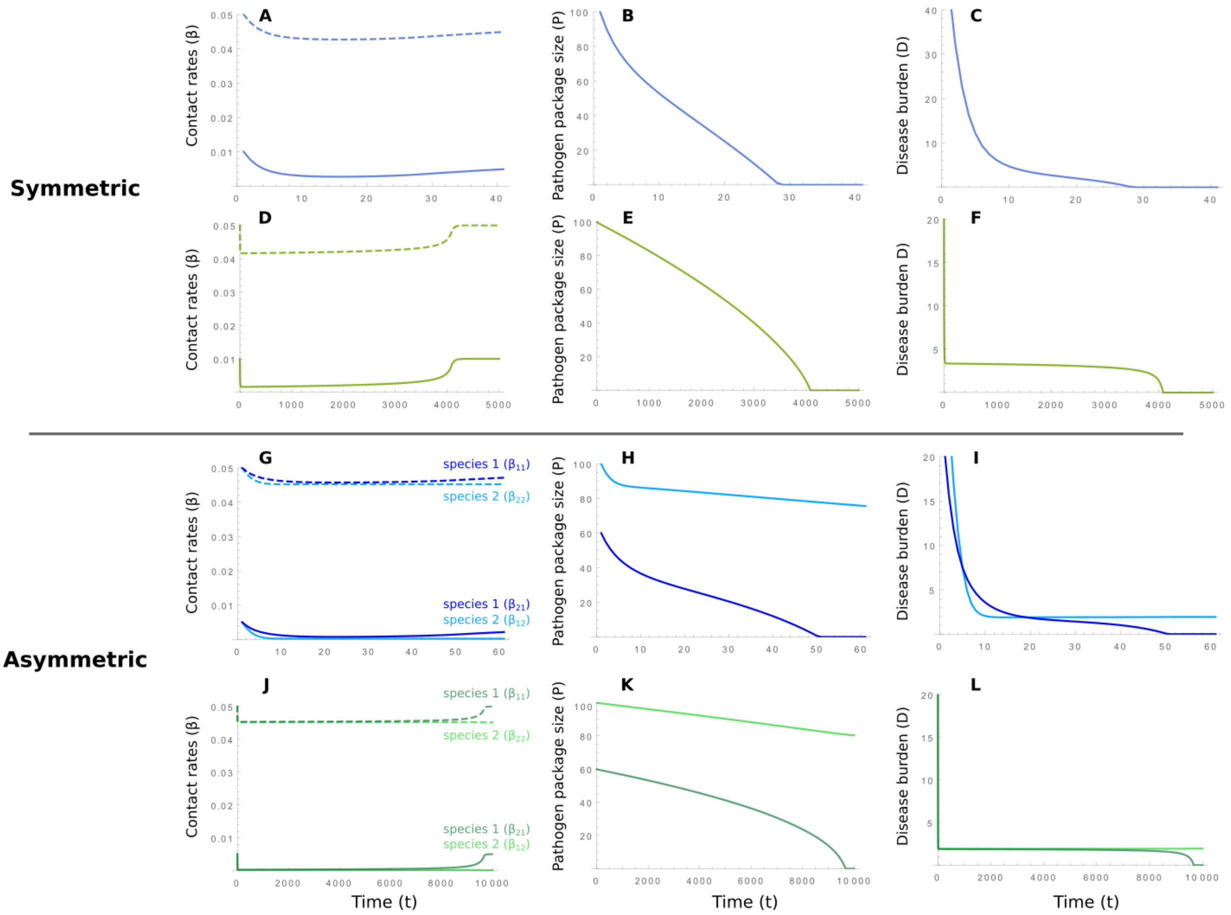

Supplementary Figure 4: Two examples of dynamics of the two-time-scales model for the introgression to disease transmission ratios (c) values marked in Supplementary Figure 3. (A)–(F) show the results for the symmetric case, comparable with Figure 3 in the main text, and (G)–(L) show the results for the asymmetric case, comparable with Figure 4 in the main text. For the panels with blue curves (corresponding to the blue line in Supplementary Figure 3), disease transmission to introgression values are $c = 1000$, while the rest of the parameters are the same as those in Figures 3 and 4 in the main text. For the panels with green curves (corresponding to the green line in Supplementary Figure 3), c values are $c = 10$, while the rest of the parameters are the same as those in Figures 3 and 4 in the main text. Notice that the times scales for the different panels are different.

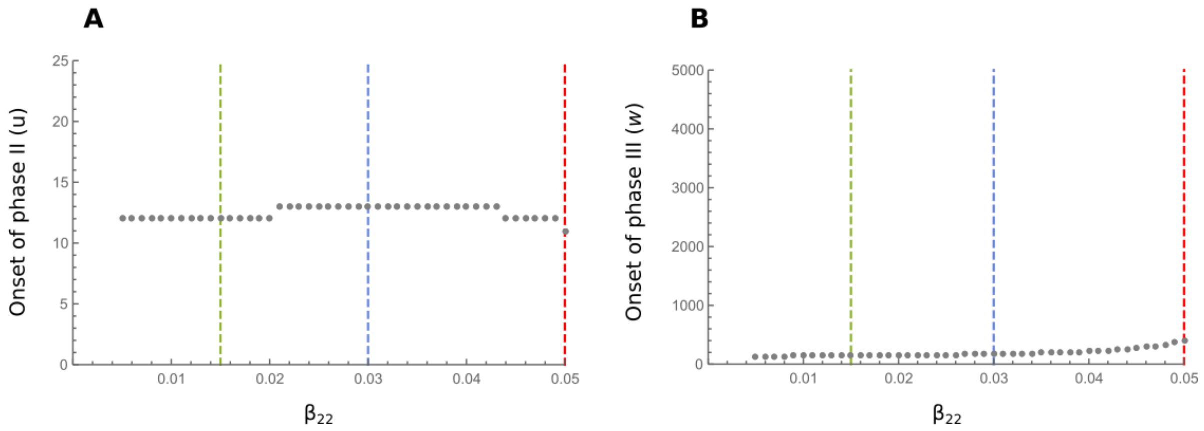

Supplementary Figure 5: Time of onset of phases II (u) and III (w) for different $\beta_{22}(0)$ values in the two-time-scales model. Dashed lines show the parameter values for which we show the dynamics explicitly, in the main text in Figures 3 (red, which is the symmetric case of $\beta_{22}(0) = \beta_{11}(0) = 0.05$), or in Supplementary Figure 6 (green and blue). (A) Onset of phase II (u). (B) Onset of phase III (w).

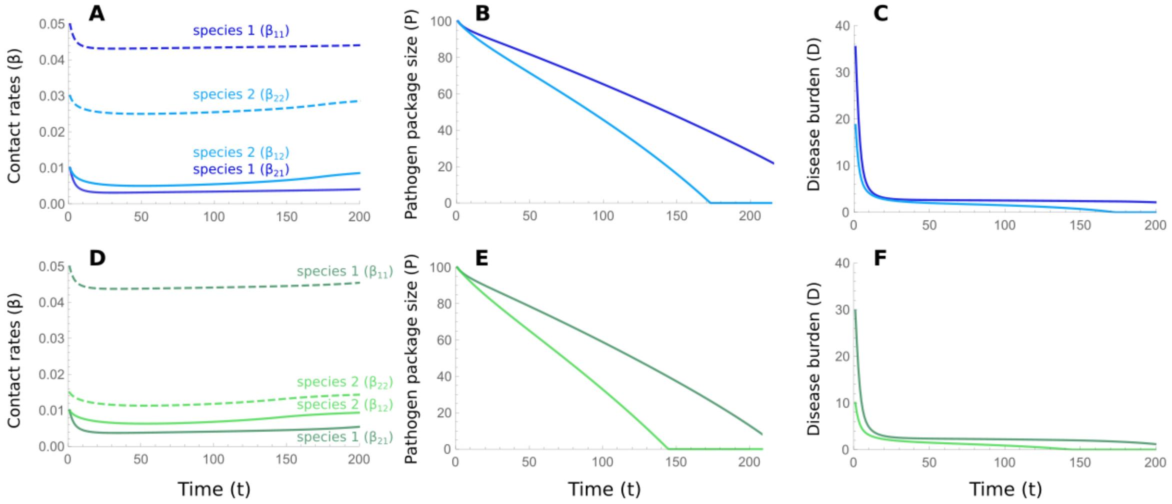

Supplementary Figure 6: Two examples of dynamics of the two-time-scales model for the $\beta_{22}(0)$ values marked in Supplementary Figure 5. The results are comparable with Figure 4 in the main text. For the panels with blue curves (corresponding to the blue line in Supplementary Figure 5), within-species contact rates in species 2 are $\beta_{22}(0) = 0.03$, while the rest of the parameters are the same as those in Figure 4 in the main text. For the panels with green curves (corresponding to the green line in Supplementary Figure 5), $\beta_{22}(0)$ values are $\beta_{22}(0) = 0.015$, while the rest of the parameters are the same as those in Figure 4 in the main text.

Single-time-scale model

For the single-time-scale model, we conduct a similar analysis for exploring the parameter ranges to the one described above for the two-times-scales model. We vary one parameter at a time, using the scenarios in Figure 5 in the main text as our baseline. We evaluate the onset of phase II (u) and phase III (w), defined in a similar manner. Since this model is defined in continuous time, u_{N_i} is defined as the earliest time in which the absolute value of the derivative of N_i is smaller than 10^{-3}. The onset of phase II, u, is defined as the maximum of u_{N_1} and u_{N_2}. The onset of phase III, w, is defined as the earliest time, t, in which the disease burden is lifted in one of the species,i.e. either $\alpha_1(t) = 0$ or $\alpha_2(t) = 0$ (w_{α_1} and w_{α_2}, respectively).

Between-species contact rates

We varied the between-species transmission rate, β_a, from 0.01 to 0.5 in increments of 0.01, for the symmetric and asymmetric cases. Supplementary Figure 7 shows the onset of the phases for different β_a values, and full dynamics for two examples ($\beta_a = 0.02$ in blue, and $\beta_a = 0.005$ in green) are shown in Supplementary Figure 8. For the symmetric case, the onset of both phases decreases as the between-species contact rates increase, since faster contact rates translate to faster dynamics in the system. In the asymmetric case, the onset of phase III decreases as β_a increases, but the onset of phase II initially increases but around $\beta_a = 0.4$ begins to increase as β_a increases. This may be due to the complex feedback between introgression, disease transmission, and population dynamics, which may result in different dynamics at different parts of the parameter space.
The between-species contact rate β_a plays a role both in determining inter-species transmission of disease (Equations (19) and (20) in the main text) and the rate of introgression (Equations (21) and (22) in the main text). In general, increase in β_a leads to faster dynamics in which the onset of both phase II and phase III occur earlier; however, for very high β_a and asymmetry in disease-related mortality rates, complex interaction in the equations lead to non-trivial changes in the onset of the phases (Supplementary Figure 7).

Ratio of introgression to disease transmission

We varied the introgression to disease transmission ratio from $c = 0.01$ to $c = 0.3$ in increments of 0.01. In the asymmetric case ($\alpha_1(0) = 0.5, \alpha_2(0) = 1$), with c values above 0.3, w becomes very low, eventually even lower than u. This is because we defined the onset of phase II, u, to be the maximum of u_{N_1} and u_{N_2}, and w to be the minimum of w_{α_1} and w_{α_2}, and with very high rates of adaptive introgression one species may overcome disease burden before the other species has stabilized.

Therefore, the notion of different phases, as discussed in the main text, does not apply to such high introgression rates. We therefore limited our range of investigation of this parameter to c values up to 0.3.
Supplementary Figure 9 shows the onset of the two phases for different c values, and two examples of the dynamics ($c = 0.01$ and $c = 0.3$) are shown in Supplementary Figure 10. The time of onset of phase II decreased as we increased c. The same is observed for the onset of phase III, due to higher c resulting in faster adaptive introgression. These results are similar to those observed for the two-time-scales model (Supplementary Figure 3). For the symmetric case, the dynamics in the examples we focused on are qualitatively similar to those in Figure 5 in the main text(Supplementary Figure 10). For the asymmetric case, the dynamics have characteristics that are different than those in Supplementary Figure 5, such as switches between whether $N_1 > N_2$ or $N_2 > N_1$, but some characteristics remain the same: high initial disease burden which rapidly decreases as population sizes decrease (phase I), followed by a relatively stable phase (phase II) with relatively constant low disease burden, followed by overcoming of disease burden of species 1 and increase in its population density (phase III).
The ratio between disease transmission and introgression, c, plays a role in Equations (21) and (22) in the main text, where higher c values lead to faster introgression. Therefore, increasing c results both in earlier onset of phase II and earlier onset of phase III (Supplementary Figure 9).

Initial asymmetry in population densities

As discussed above for the two-time-scales model, a plausible source of asymmetry, other than the characteristics of the pathogen package, is population densities. We therefore explored this source of asymmetry by considering the symmetric case in Figure 5 in the main text as our baseline, and varying the initial population density of species 2, $N_2(0)$ (initial population density of species 1 was kept as $N_1(0) = 2$). We kept the intrinsic demographic parameters, K, λ, and μ the same for both species. We varied the ratios between the two population densities from $N_2(0) = 0.01 \times N_1(0)$ to $N_2(0) = 1 \times N_1(0)$ in increments of 0.01.
Supplementary Figure 11 shows the onset of the two phases for different ratios of initial population densities. Except for very high asymmetry, with species 1 being more than 20 times more dense than species 2, the onset of phase II decreased with more asymmetry. The onset of phase III decreased as the asymmetry was stronger. The examples in Supplementary Figure 12 are qualitatively similar to those in Figure 5 in the main text, with the three phases observable.
As with the two-time-scale model, for asymmetry in population density ($\beta_{22}(0)$ in the two-time-scales model, which could be interpreted as population density), the species that started out with the lower density, species 2, is the one that eventually overcomes

disease burden faster, and consequently becomes denser than the other species.
The intial density of species 2, $N_2(0)$, plays a role in the initial conditions of Equations (17) and (18) in the main text. When the initial population density in species 2 is decreased, it has less effect on species in terms of infections (Equations (17) and (18) in the main text). This results in species 1 initially maintaining higher population densities with lower $N_2(0)$ values (Supplementary Figure 12). This, in turns, results in higher rates of introgression in species 2 with lower $N_2(0)$, leading to earlier onset of phase III (Supplementary Figure 11). The onset of phase II is not simply determined with changes of $N_2(0)$.

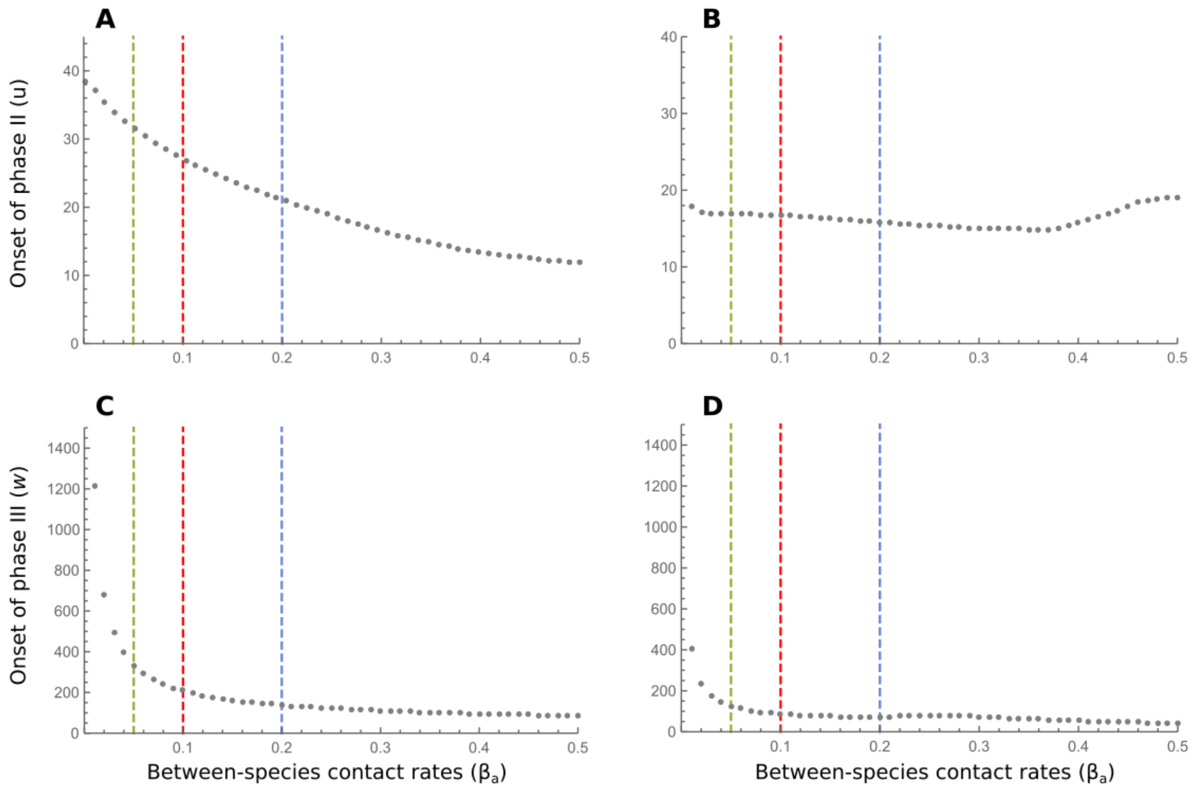

Supplementary Figure 7: Time of onset of phases II (u) and III (w) for different β_a values in the single-time-scales model. Dashed lines show the parameter values for which we show the dynamics explicitly, in the main text in Figure 5 (red), or in Supplementary Figure 8 (green and blue). (A) Onset of phase II for the symmetric case. (B) Onset of phase II for the asymmetric case ($\alpha_1(0) = 0.5$, $\alpha_2(0) = 1$). (C) Onset of phase III for the symmetric case. (D) Onset of phase III for the asymmetric case ($\alpha_1(0) = 0.5$, $\alpha_2(0) = 1$). Other than the the parameter β_a, all other parameters are the same as those in Figure 5 in the main text.

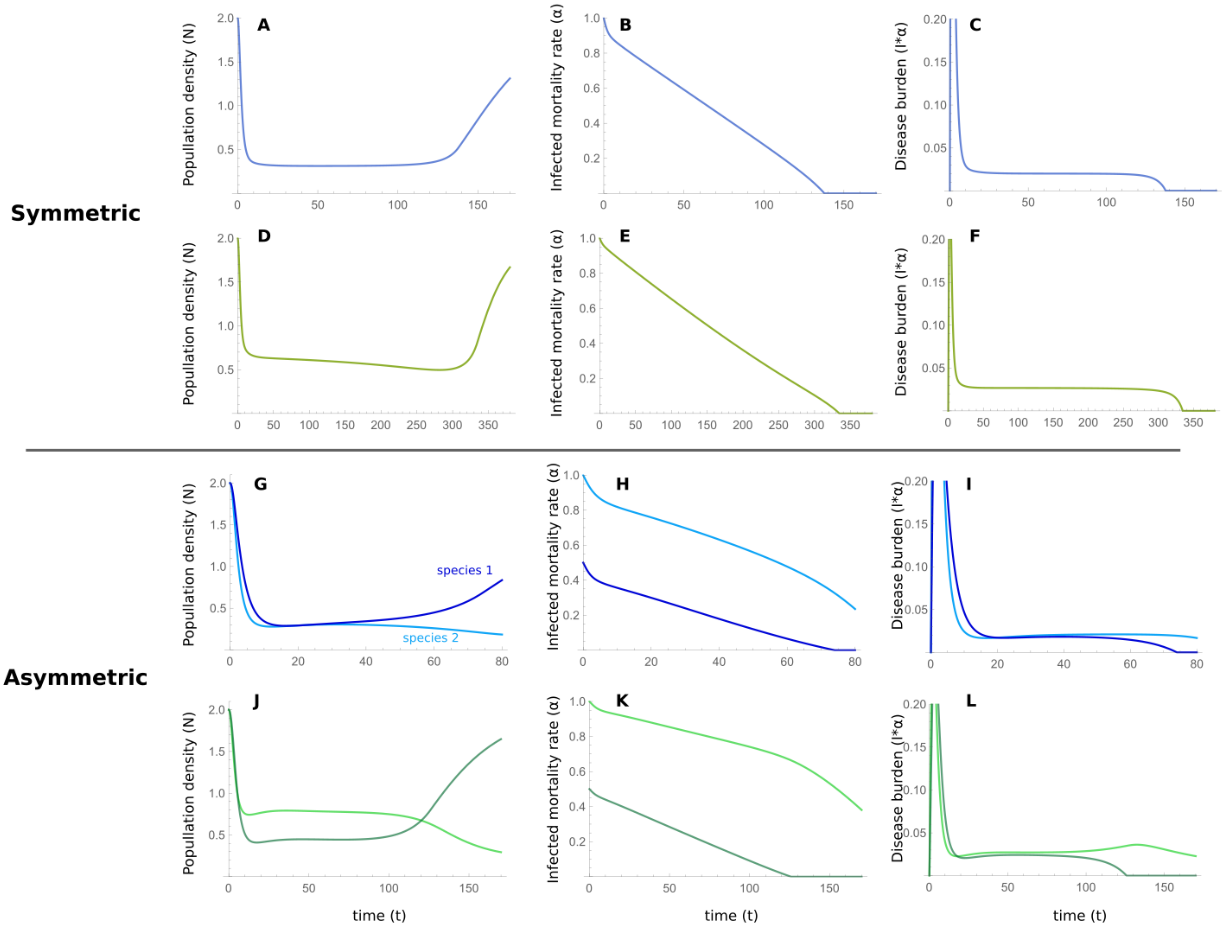

Supplementary Figure 8: Two examples of dynamics of the single-time-scale model for the β_a values marked in Supplementary Figure 7. (A)–(F) show the results for the symmetric case and (G)–(L) show the results for the asymmetric ($\alpha_1(0) = 0.5$, $\alpha_2(0) = 1$), comparable with Figure 5 in the main text. For the panels with blue curves (corresponding to the blue line in Supplementary Figure 7), between-species contact rates are $b_a = 0.2$, while the rest of the parameters are the same as those in Figure 5 in the main text. For the panels with green curves (corresponding to the green line in Supplementary Figure 7), between-species contact rates are $b_a = 0.05$, while the rest of the parameters are the same as those in Figure 5 in the main text. Notice that the times scales for the different panels are different.

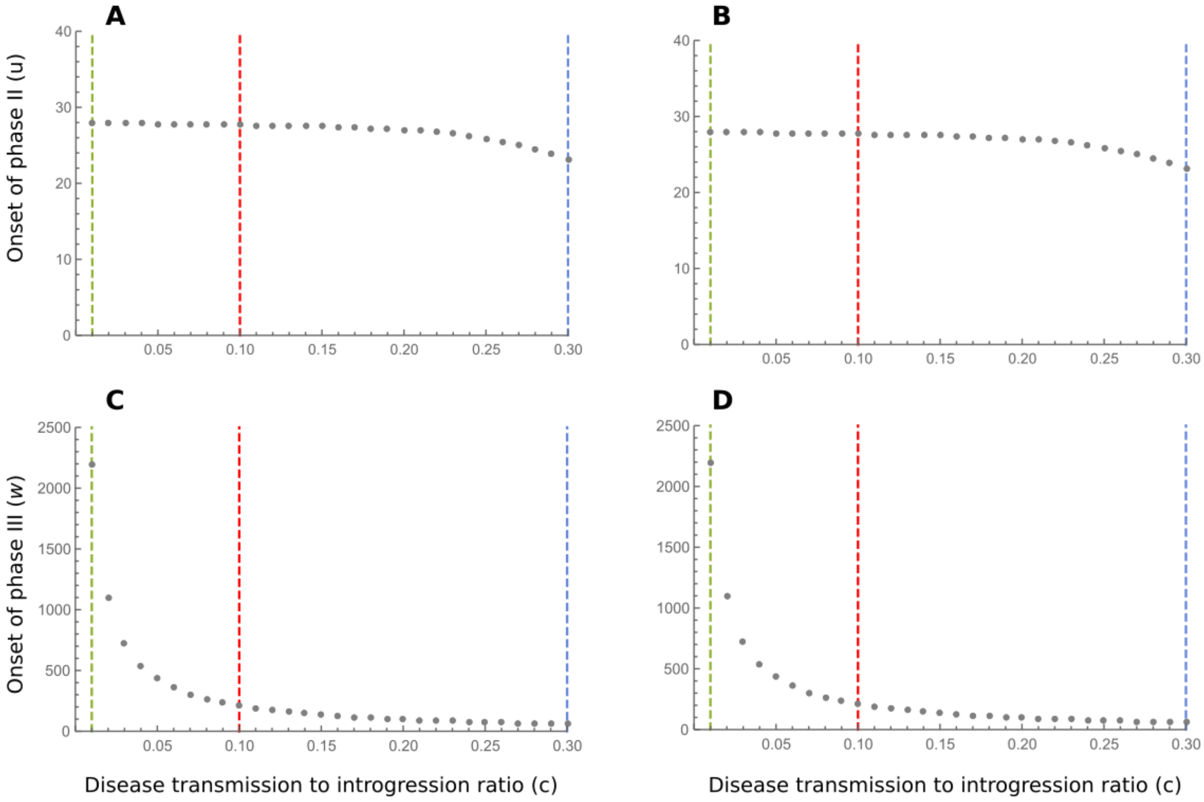

Supplementary Figure 9: Time of onset of phases II (u) and III (w) for different c values in the single-time-scales model. Dashed lines show the parameter values for which we show the dynamics explicitly, in the main text in Figure 5 (red), or in Supplementary Figure 10 (green and blue). (A) Onset of phase II for the symmetric case. (B) Onset of phase II for the asymmetric case ($\alpha_1(0) = 0.5$, $\alpha_2(0) = 1$). (C) Onset of phase III for the symmetric case. (D) Onset of phase III for the asymmetric case ($\alpha_1(0) = 0.5$, $\alpha_2(0) = 1$). Other than the parameter c, all other parameters are the same as those in Figure 5 in the main text.

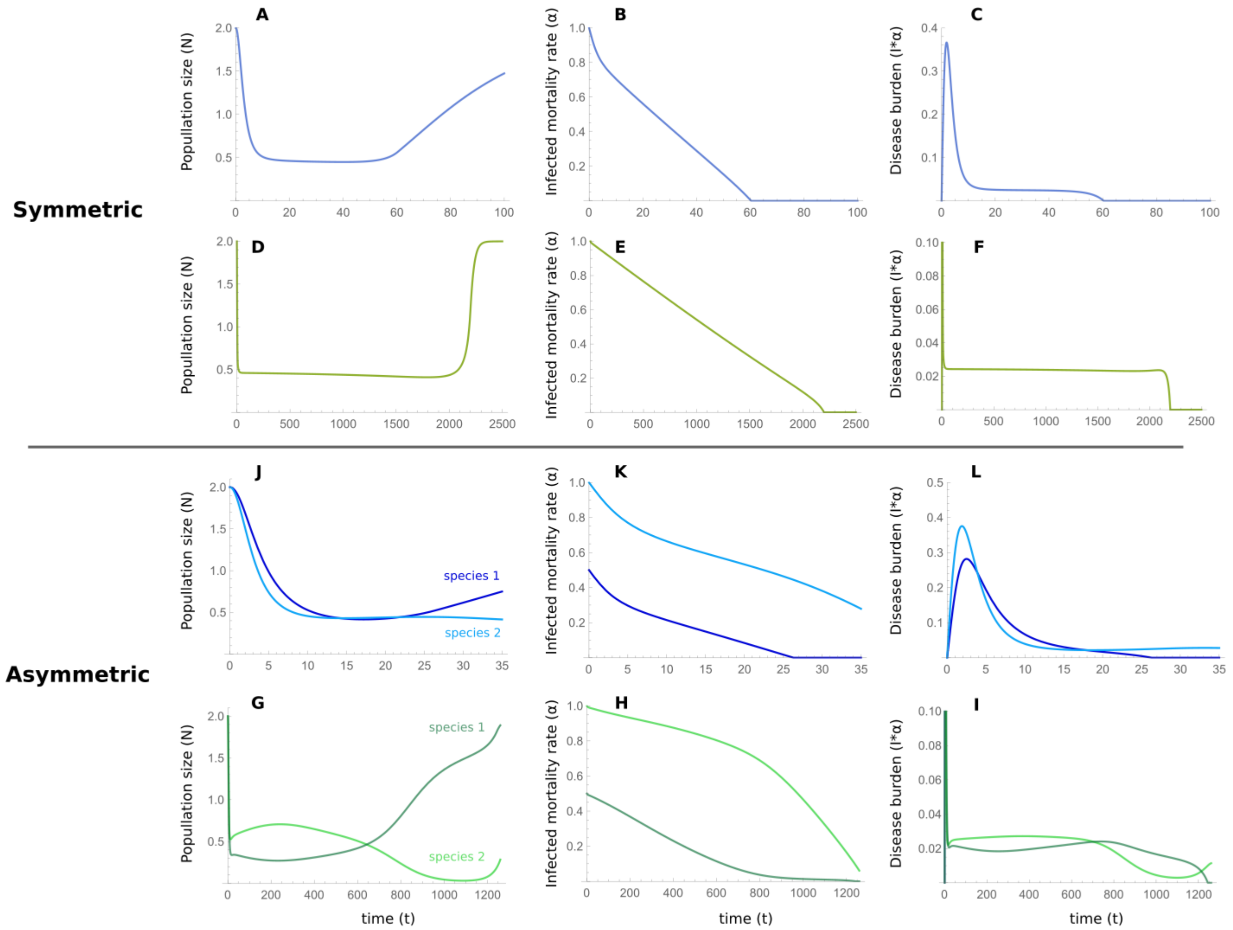

Supplementary Figure 10: Two examples of dynamics of the single-time-scale model for the c values marked in Supplementary Figure 9. (A)–(F) show the results for the symmetric case and (G)–(L) show the results for the asymmetric case ($\alpha_1(0) = 0.5$, $\alpha_2(0) = 1$), comparable with Figure 5 in the main text. For the panels with blue curves (corresponding to the blue line in Supplementary Figure 9), $c = 0.3$, while the rest of the parameters are the same as those in Figure 5 in the main text. For the panels with green curves (corresponding to the green line in Supplementary Figure 9), $c = 0.01$, while the rest of the parameters are the same as those in Figure 5 in the main text. Notice that the times scales for the different panels are different.

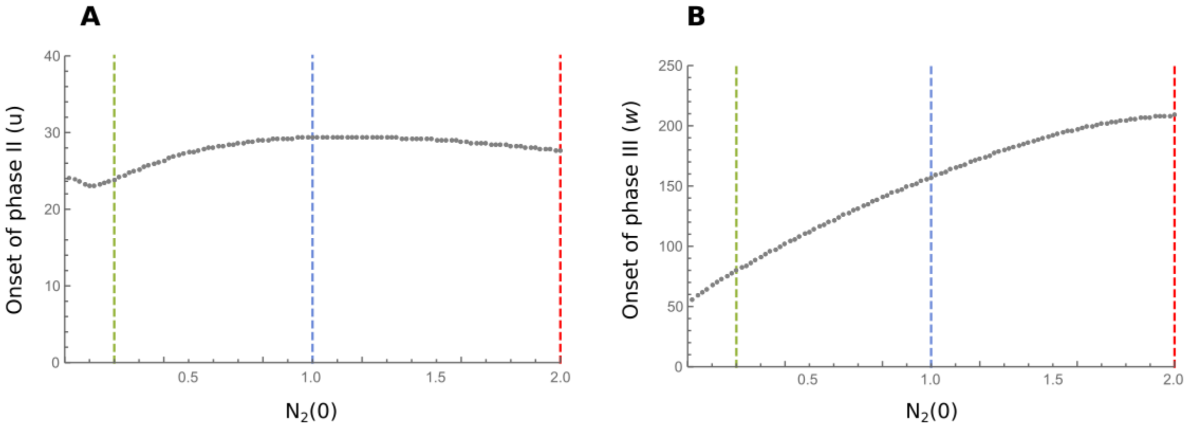

Supplementary Figure 11: Time of onset of phases II (u) and III (w) for different $N_2(0)$ values in the single-time-scales model. Dashed lines show the parameter values for which we show the dynamics explicitly, in the main text in Figures 5 (red), or in Supplementary Figure 12 (green and blue). (A) Onset of phase II (u). (B) Onset of phase III (w).

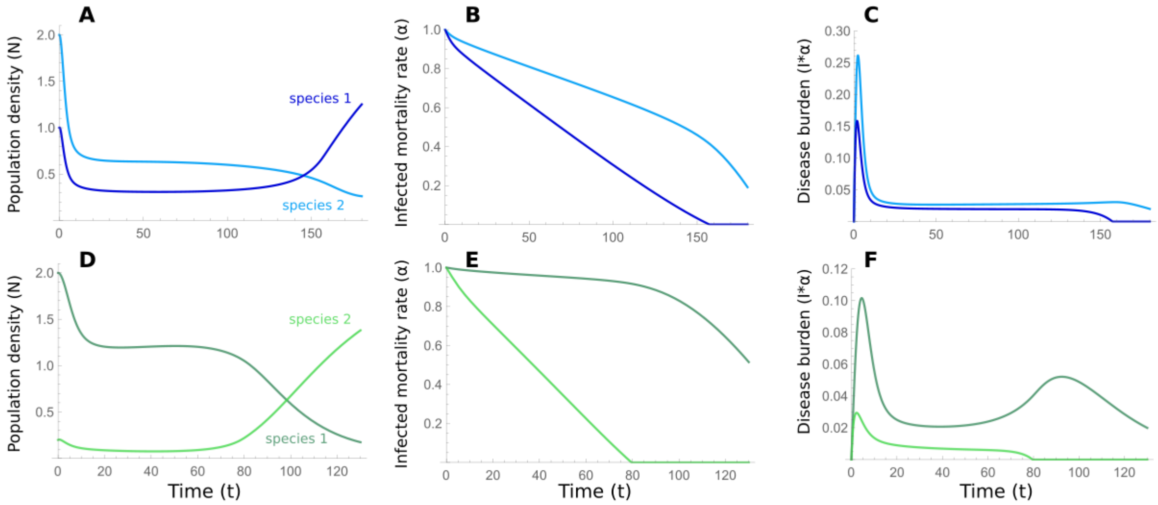

Supplementary Figure 12: Two examples of dynamics of the single-time-scale model for the $N_2(0)$ values marked in Supplementary Figure 11. The results are comparable with Figure 5 in the main text. For the panels with blue curves (corresponding to the blue line in Supplementary Figure 11), $N_2(0) = 0.5$, while the rest of the parameters are the same as those in Figure 5 in the main text. For the panels with green curves (corresponding to the green line in Supplementary Figure 11), $N_2(0) = 0.1$, while the rest of the parameters are the same as those in Figure 5 in the main text. Notice that the times scales for the different panels are different.

Supplementary Note 2 — Modeling regular adaptation in addition to adaptive introgression

In this section we explore adding adaptation to disease due to de novo immune-related mutations in both species, in addition to introgression.

Two-time-scales model

In the two-time-scales model, adaptation to the novel pathogen package is measured in terms of the number of pathogens to which each of the species is vulnerable. While introgression depends on inter-species contact rates, regular within-species adaptation is expected to be independent of these. In many cases, adaptations are acquired by natural selection acting on newly arisen mutations or on standing variation. The process by which this occurs depends on several factors, including the strength of selection, the effective population size and genetic drift, and the rate of mutation.
Modeling the population genetics of this process in the context of our model is not a simple task. We therefore decided to make many simplifying assumption, and instead of incorporating a population genetic model, we assume that beneficial immune-related mutations, which reduce the experienced pathogen package size P, appear and are driven to fixation at a constant rate (see [1–6] for similarly simplified models). Moreover, although fixation through selection is not an instantaneous process, and in intermediate steps the population would be composed of both individuals carrying the immune-related alleles and those that do not carry them, we assume that the time between appearance and fixation is negligible at the evolutionary time scale. These assumptions, which may not be appropriate in many cases, allow us to model regular adaptation as a single parameter, μ_a, which is the (constant) rate at which immune-related mutations that are eventually driven to fixation in the population convey tolerance to a single pathogen. This rate is measured according to the evolutionary time-scale of this model (t). Therefore, at each time step t, species i evolves tolerance to $\mu_a P_i(t)$ novel pathogens (with the assumption that the rate of evolution of tolerance to each pathogen is the same, and that the evolutionary processes for each pathogen are independent). We also assume that these rates are the same for both species.
The equation describing the pathogen package size is a modified version of Equations (21) and (22) in the main text, namely :

$$P_1(t) = \max\left\{(1-\mu_a)P_1(t-1) - c\,\beta_{21}(t-1), 0\right\} \tag{1}$$

$$P_2(t) = \max\left\{(1-\mu_a)P_2(t-1) - c\,\beta_{12}(t-1), 0\right\}\,, \tag{2}$$

and the two-time-scales model with regular adaptation is identical to the one described in the main text but with Supplementary Equations (1) and (2) replacing Equations (14) and (15) in the main text.
For this model, we varied the μ_a parameter from 0 to 0.05 in increments of 0.001, with the other parameters remaining fixed to the baseline values of Figure 3 in the main text. We used the same definition of time of onset of phases II and III as presented in Appendix A to explore the effect of regular adaptation on the phases (Supplementary Figure 13). We also show two examples of the dynamics ($\mu_a = 0.005$ in green and $\mu_a = 0.01$ in blue) in Supplementary Figure 14.
The examples in Supplementary Figure 14 demonstrate that the dynamics are qualitatively similar to those without regular adaptation, for the parameters we investigated, in the sense that the three phases can be observed. In Supplementary Figure 13, it can be seen that the onset of phase II is almost unchanged with increased regular adaptation, with a slight decrease, but the onset of phase III is earlier as the level of regular adaptation increases. This is so since in our model, we added regular adaptation in addition to adaptive introgression, in essence modeling two pathways by which tolerance can be evolved, and therefore the species overcome disease burden sooner than with adaptive introgression alone.

Single-time-scale model

We also incorporated regular adaptation in the single time-scale model, with the same simplifying assumption and the same reservations. Here, we introduce a parameter μ_a, which is the constant rate of appearance of mutations that convey tolerance to pathogens, and which are eventually driven to fixation. Since we model population densities explicitly in this model, and the rate of appearance of novel mutations in the populations is proportional to population size, we model the effect of regular adaptation as a reduction of disease-induced mortality, α_i, by the rate of *de novo* appearance of adaptive mutations in the population, $N_i\mu_b$:

$$\frac{d\alpha_1(t)}{dt} = -c\beta_a N_2(t) - \mu_b N_1(t) \quad \text{provided } \alpha_1(t) > 0\text{, otherwise } \frac{d\alpha_1(t)}{dt} = 0 \quad (3)$$
$$\frac{d\alpha_2(t)}{dt} = -c\beta_a N_1(t) - \mu_b N_2(t) \quad \text{provided } \alpha_2(t) > 0\text{, otherwise } \frac{d\alpha_2(t)}{dt} = 0\,. \quad (4)$$

The single-time-scale model with regular adaptation is the same as in the main text except that Supplementary Equations (3) and (4) replacing Equations (21) and (22) in the main text.
We varied the μ_b parameter from 0 to 0.05 in increments of 0.001, with the other parameters remaining fixed to the baseline values of Figure 5 in the main text (symmetric case). We used the same definition of time of onset of phases II and III as

presented in Appendix A to explore the effect of regular adaptation on the phases (Supplementary Figure 15). We also show two examples of the dynamics ($\mu_a = 0.005$ in green and $\mu = 0.01$ in blue) in Supplementary Figure 16.
As with the two-time-scales model, the examples for different rates of μ_b show similar dynamics with the three phases described in the main text (Supplementary Figure 16). For higher rates of regular adaptation, the onset of the second phase does not change much in the parameter range range we investigated, but the onset of phase II is significantly reduced (Supplementary Figure 15). As was discussed for the two-time-scales model, we model regular adaptation as a process which occurs in parallel and in addition to adaptive introgression, and therefore disease burden is overcome more rapidly with more regular adaptation.

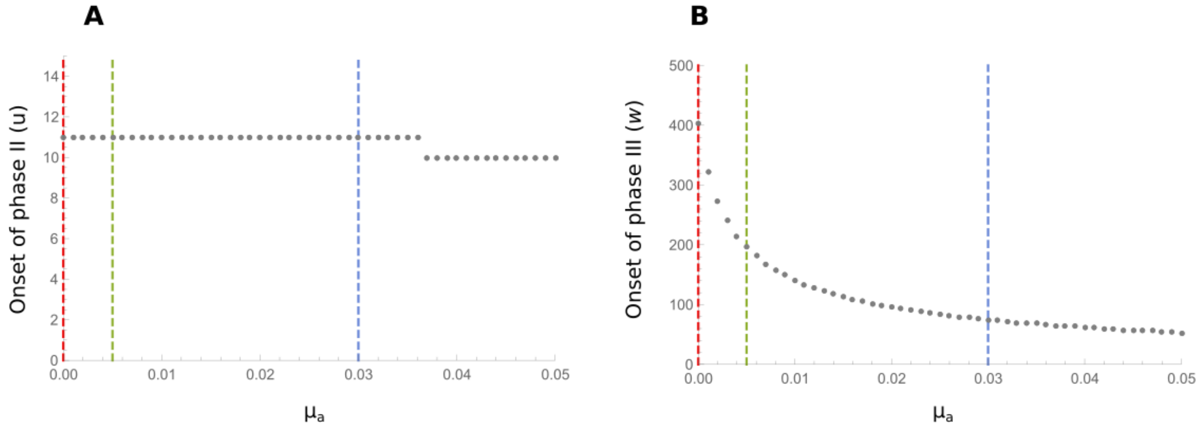

Supplementary Figure 13: Time of onset of phases II (u) and III (w) for different μ_a values in the two-time-scales model with regular adaptation. Dashed lines show the parameter values for which we show the dynamics explicitly, in the main text in Figures 3 (red), or in Supplementary Figure 14 (green and blue). (A) Onset of phase II (u). (B) Onset of phase III (w).

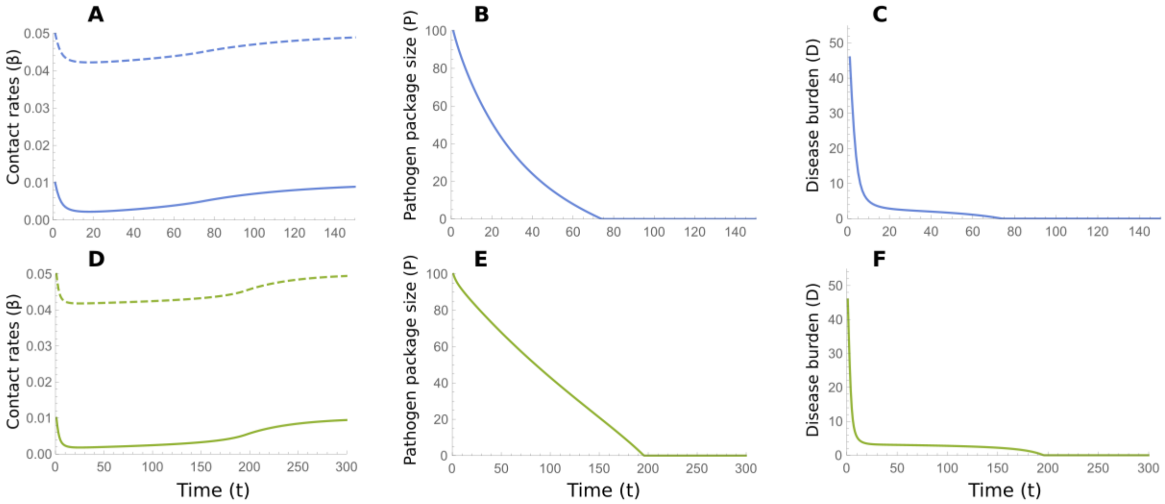

Supplementary Figure 14: Two examples of dynamics of the two-time-scales model with regular adaptation for the values marked in Supplementary Figure 13. For the panels with blue curves (corresponding to the blue line in Supplementary Figure 13), $\mu_a = 0.03$, while the rest of the parameters are the same as those in Figure 3 in the main text. For the panels with green curves (corresponding to the green line in Supplementary Figure 13), $\mu_a = 0.0005$, while the rest of the parameters are the same as those in Figure 3 in the main text. Notice that the times scales for the different panels are different.

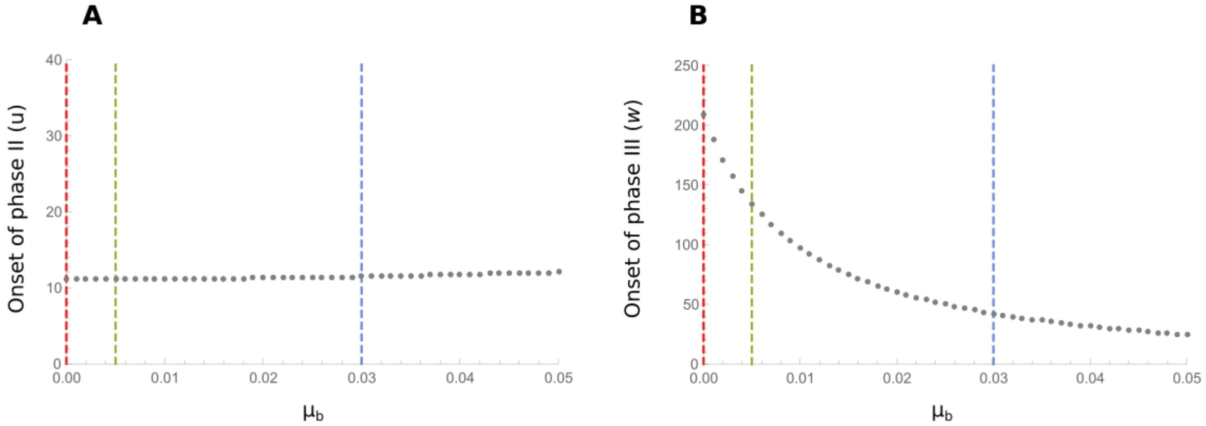

Supplementary Figure 15: Time of onset of phases II (u) and III (w) for different μ_b values in the single-time-scale model with regular adaptation. Dashed lines show the parameter values for which we show the dynamics explicitly, in the main text in Figures 5 (red), or in Supplementary Figure 16 (green and blue). (A) Onset of phase II (u). (B) Onset of phase III (w).

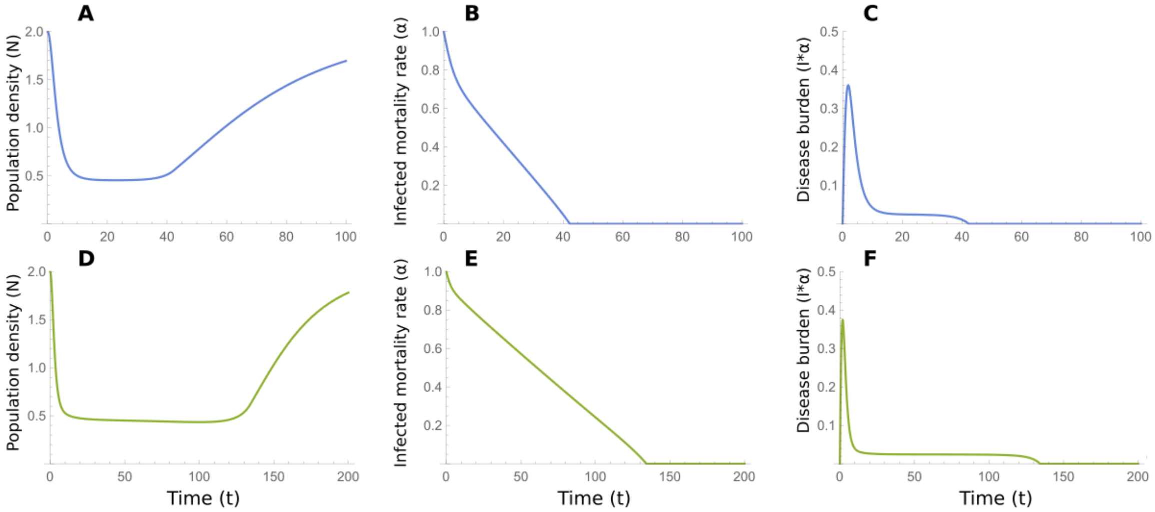

Supplementary Figure 16: Two examples of dynamics of the single-time-scale model with regular adaptation for the values marked in Supplementary Figure 15. For the panels with blue curves (corresponding to the blue line in Supplementary Figure 15), $\mu_a = 0.03$, while the rest of the parameters are the same as those in Figure 5 in the main text(symmetric case). For the panels with green curves (corresponding to the green line in Supplementary Figure 15), $\mu_a = 0.0005$, while the rest of the parameters are the same as those in Figure 5 in the main text (symmetric case). Notice that the times scales for the different panels are different.

Supplementary Note 3 — Modeling inter-species competition

Given the physical similarity and close phylogenetic relations of Neanderthals and Moderns, it is plausible that when they interacted in the Levant they were in competition for similar resources, and that they occupied overlapping ecological niches [7, 8]. In the models presented in the main text, we did not incorporate direct competition between the species. In the two-time-scales model, where demography is only implicitly modeled, incorporating competition would present some difficulty. However, in the single-time-scale model, demographics are explicitly modeled, and therefore, it is easier to accommodate competition between the species in the model, and explore how that might affect the dynamics we describe. In this section we relax the assumption of no competition in the single-time-scale model, and incorporate the effect of competition on growth rates.
For this purpose, we introduce a competition parameter ϕ, which measures the niche overlap of the two species, i.e. for $\phi = 0$, the species' niches do not overlap and there is no competition, and for $\phi = 1$, the niches are identical and inter-species competition is the same as within-species competition [9]. In the main text, we modeled density-dependent growth rate as a function of the intrinsic growth rate λ and the half-maximum population density K, and now we incorporate inter-species competition:

$$\Lambda_i(N_i, N_j) = \frac{\lambda K}{K + (N_i + \phi N_j)}, \quad i \neq j, \; i,j = 1,2. \tag{5}$$

The single-time-scale model with competition is, therefore, the model described in the main text with Supplementary Equation (5) used instead of Equation (16) in the main text (for $\phi = 0$, this model converges to the single-time-scale model presented in the main text).
With increased competition between the species, the onset of phase II is slightly delayed, and the onset of phase III is more substantially delayed (Supplementary Figure 17). Therefore, competition would act to increase the duration of the second stable phase, for the parameters we explored (Supplementary Figure 18). For the two examples we investigated, the dynamics are qualitatively similar to those presented in the main text, as the three phases can be observed.

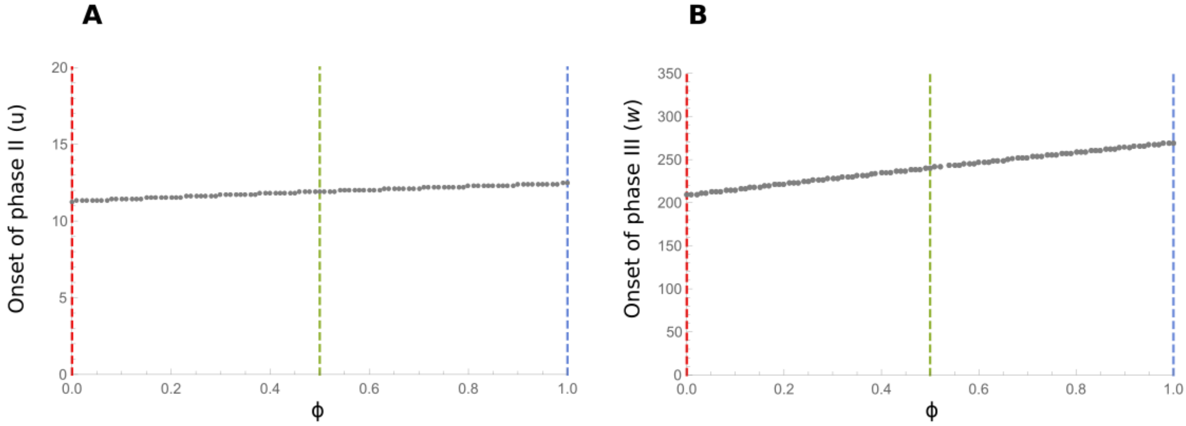

Supplementary Figure 17: Time of onset of phases II (u) and III (w) for different ϕ values in the single-time-scale model with competition. Dashed lines show the parameter values for which we show the dynamics explicitly, in the main text in Figures 5 (red), or in Supplementary Figure 18 (green and blue). (A) onset of phase II (u). (B) Onset of phase III (w).

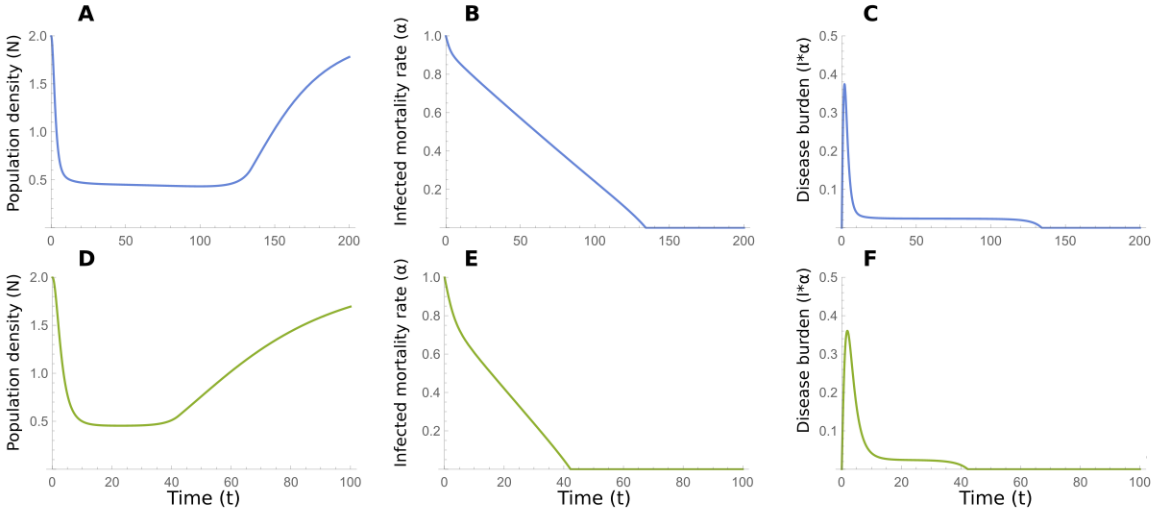

Supplementary Figure 18: Two examples of dynamics of the single-time-scale model with competition for the values marked in Supplementary Figure 17. For the panels with blue curves (corresponding to the blue line in Supplementary Figure 17), $\phi = 1$ (full niche overlap), while the rest of the parameters are the same as those in Figure 5 in the main text (symmetric case). For the panels with green curves (corresponding to the green line in Supplementary Figure 17), $\phi = 0.5$ (partial niche overlap), while the rest of the parameters are the same as those in Figure 5 in the main text (symmetric case). Notice that the times scales for the different panels are different.

Supplementary References

1. Orr, H. A. The population genetics of adaptation: the adaptation of dna sequences. *Evolution* **56,** 1317–1330 (2002).
2. Gillespie, J. *Population genetics: a concise guide* (The Johns Hopkins University Press, Baltimore, Mariland).
3. Ewens, W. *Mathematical Population Genetics: I. Theoretical Introduction* second (Springer, New York, NY, 2004).
4. Coop, G. & Ralph, P. Patterns of neutral diversity under general models of selective sweeps. *Genetics* **192,** 205–224 (2012).
5. Messer, P. W. & Petrov, D. A. Population genomics of rapid adaptation by soft selective sweeps. *Trends in Ecology and Evolution* **28,** 659–669 (2013).
6. Kolodny, O., Creanza, N. & Feldman, M. W. Evolution in leaps: The punctuated accumulation and loss of cultural innovations. *Proceedings of the National Academy of Sciences* **112,** E6762–E6769 (2015).
7. Flores, J. C. A mathematical model for Neanderthal extinction. *Journal of Theoretical Biology* **191,** 295–298 (1998).
8. Flores, J. C. Diffusion coefficient of Modern Humans outcompeting Neanderthals. *Journal of Theoretical Biology* **280,** 189–190 (2011).
9. Getz, W. M. Metaphysiological and evolutionary dynamics of populations exploiting constant and interactive resources:R-K selection revisited. *Evolutionary Ecology* **7,** 287–305 (1993).
